# *In-situ* synthesis of quantum dots in the nucleus of live cells

**DOI:** 10.1093/nsr/nwae021

**Published:** 2024-01-12

**Authors:** Yusi Hu, Zhi-Gang Wang, Haohao Fu, Chuanzheng Zhou, Wensheng Cai, Xueguang Shao, Shu-Lin Liu, Dai-Wen Pang

**Affiliations:** State Key Laboratory of Medicinal Chemical Biology, Frontiers Science Centre for New Organic Matter, Tianjin Key Laboratory of Biosensing and Molecular Recognition, Research Centre for Analytical Sciences, College of Chemistry, School of Medicine, and Frontiers Science Centre for Cell Responses, Nankai University, Tianjin 300071, China; Haihe Laboratory of Sustainable Chemical Transformations, Tianjin 300192, China; State Key Laboratory of Medicinal Chemical Biology, Frontiers Science Centre for New Organic Matter, Tianjin Key Laboratory of Biosensing and Molecular Recognition, Research Centre for Analytical Sciences, College of Chemistry, School of Medicine, and Frontiers Science Centre for Cell Responses, Nankai University, Tianjin 300071, China; Haihe Laboratory of Sustainable Chemical Transformations, Tianjin 300192, China; State Key Laboratory of Medicinal Chemical Biology, Frontiers Science Centre for New Organic Matter, Tianjin Key Laboratory of Biosensing and Molecular Recognition, Research Centre for Analytical Sciences, College of Chemistry, School of Medicine, and Frontiers Science Centre for Cell Responses, Nankai University, Tianjin 300071, China; Haihe Laboratory of Sustainable Chemical Transformations, Tianjin 300192, China; State Key Laboratory of Medicinal Chemical Biology, Frontiers Science Centre for New Organic Matter, Tianjin Key Laboratory of Biosensing and Molecular Recognition, Research Centre for Analytical Sciences, College of Chemistry, School of Medicine, and Frontiers Science Centre for Cell Responses, Nankai University, Tianjin 300071, China; State Key Laboratory of Medicinal Chemical Biology, Frontiers Science Centre for New Organic Matter, Tianjin Key Laboratory of Biosensing and Molecular Recognition, Research Centre for Analytical Sciences, College of Chemistry, School of Medicine, and Frontiers Science Centre for Cell Responses, Nankai University, Tianjin 300071, China; Haihe Laboratory of Sustainable Chemical Transformations, Tianjin 300192, China; State Key Laboratory of Medicinal Chemical Biology, Frontiers Science Centre for New Organic Matter, Tianjin Key Laboratory of Biosensing and Molecular Recognition, Research Centre for Analytical Sciences, College of Chemistry, School of Medicine, and Frontiers Science Centre for Cell Responses, Nankai University, Tianjin 300071, China; Haihe Laboratory of Sustainable Chemical Transformations, Tianjin 300192, China; State Key Laboratory of Medicinal Chemical Biology, Frontiers Science Centre for New Organic Matter, Tianjin Key Laboratory of Biosensing and Molecular Recognition, Research Centre for Analytical Sciences, College of Chemistry, School of Medicine, and Frontiers Science Centre for Cell Responses, Nankai University, Tianjin 300071, China; Haihe Laboratory of Sustainable Chemical Transformations, Tianjin 300192, China; State Key Laboratory of Medicinal Chemical Biology, Frontiers Science Centre for New Organic Matter, Tianjin Key Laboratory of Biosensing and Molecular Recognition, Research Centre for Analytical Sciences, College of Chemistry, School of Medicine, and Frontiers Science Centre for Cell Responses, Nankai University, Tianjin 300071, China; Haihe Laboratory of Sustainable Chemical Transformations, Tianjin 300192, China

**Keywords:** glutathione, live cell, nucleus, quantum dot, intracellular synthesis

## Abstract

The cell nucleus is the main site for the storage and replication of genetic material, and the synthesis of substances in the nucleus is rhythmic, regular and strictly regulated by physiological processes. However, whether exogenous substances, such as nanoparticles, can be synthesized *in situ* in the nucleus of live cells has not been reported. Here, we have achieved *in-situ* synthesis of CdS*_x_*Se_1−_*_x_* quantum dots (QDs) in the nucleus by regulation of the glutathione (GSH) metabolic pathway. High enrichment of GSH in the nucleus can be accomplished by the addition of GSH with the help of the Bcl-2 protein. Then, high-valence Se is reduced to low-valence Se by glutathione-reductase-catalyzed GSH, and interacts with the Cd precursor formed through Cd and thiol-rich proteins, eventually generating QDs in the nucleus. Our work contributes to a new understanding of the syntheses of substances in the cell nucleus and will pave the way for the development of advanced ‘supercells’.

## Teaser

This study strategically exploits cellular metabolic pathways to synthesize fluorescent quantum dots within the nucleus of live cells, pioneering the new field of synthetic biology.

## INTRODUCTION

As the largest and most important organelle in eukaryotic cells, the nucleus is the main site for the storage, replication and transcription of genetic material, playing a central role in cell metabolism, growth, differentiation, proliferation and other life activities [1]. The nuclear membrane separates the material within the nucleoplasm from the cytoplasm, which selectively controls the exchange of materials and information inside and outside the nucleus. Thus, the synthesis of substances within the nucleus is usually rhythmic, regular and under tight regulation according to physiological needs. Many studies have reported on the synthesis and related mechanisms of key biological substances in the nucleus [2,3]. However, the *in-situ* synthesis of nanoparticles in the nucleus has not yet been reported, let alone the associated regulatory mechanisms of this synthesis.

Introducing inorganic semiconductor nanocrystals—quantum dots (QDs) with excellent optical properties—into live cells via *in-situ* synthesis can hopefully construct supercells or smart engineered biosystems. Our group has systematically developed the synthesis of QDs and other nanomaterials by coupling intracellular biochemical reactions in an appropriate spatial and temporal sequence in live fungal, bacterial and even mammalian cells [4–8]. In 2009, we first achieved artificially regulated space-time-coupled live-cell synthesis of color-tunable CdSe QDs in live yeast cells. The Na_2_SeO_3_ and CdCl_2_ generated reactive Se- and Cd-precursors, and by combining them with glutathione (GSH) and related enzymes, the CdSe QDs were synthesized in live cells [4]. The genetic knockout experiments demonstrated that the GSH metabolic pathway is important for the synthetic yield of CdSe QDs, and can be manipulated to enhance the synthetic yield of CdSe QDs in transgenic yeast [6]. Meanwhile, QDs can also be synthesized in *Staphylococcus aureus* (*S. aureus*) cells, and the resulting fluorescent cells can be transformed into probes for pathogen detection [7]. Recently, synthesis of QDs in mammalian cells has been achieved, and conveniently labels the microvesicles with QDs, realizing designer cell-self-implemented labeling of microvesicles *in situ* with the intracellular-synthesized QDs [9]. Thus, if QDs can be synthesized at a specific organelle in live cells, such as the cell nucleus, smart engineered biosystems could potentially be constructed.

GSH has many important cellular functions as it forms the center of the complex antioxidant network of the cell, buffers reactive oxygen species to promote cellular redox signaling, and controls cell growth, development and defense [10]. The translocation of GSH between different compartments within the cell is essential for the regulation of cellular life activities. In particular, the recruitment and sequestration of GSH in the nucleus have profound effects on redox homeostasis and gene expression in the cell [11]. Here, we achieved *in-situ* synthesis of QDs in the nucleus by regulating the spatiotemporal coupling strategy and GSH metabolic pathway, which enriches the engineered biosynthetic system of fluorescent nanocrystals and provides key insights into the mechanism of synthesis of endogenous substances in the nucleus, contributing to a new understanding of intranuclear synthetic functions.

## RESULTS

### Enhancement of fluorescence in the nucleus with GSH treatment

**Figure 1.**
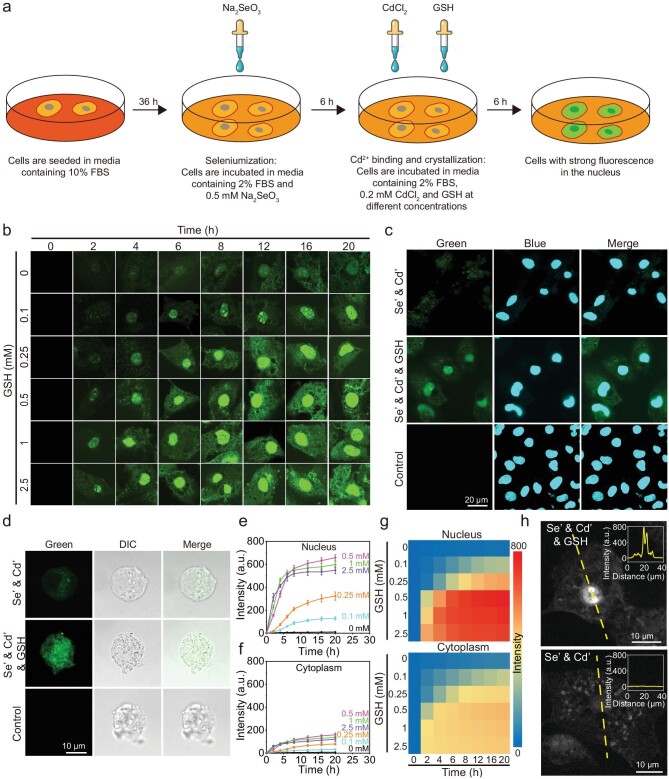
Enhanced fluorescence in the nucleus after the addition of GSH. (a) The procedure for generating fluorescent cells by treatment with sodium selenite (Na_2_SeO_3_), cadmium chloride (CdCl_2_) and glutathione (GSH). (b) Time-lapse fluorescence images of Madin-Darby canine kidney (MDCK) cells treated with Se’ and Cd’ and different concentrations of GSH. (c) Fluorescence images of the treated cells stained with nucleus dye (the first column), and Hoechst 33342 (the second column). (d) The fluorescence and differential interference contrast (DIC) images of a nucleus extracted from treated cells. (e, f) The fluorescence intensity of the nucleus (e) and cytoplasm (f) of cells treated with Se’ and Cd’ and different concentrations of GSH. (g) A heat map of the fluorescence intensity of the nucleus and cytoplasm in (e) and (f). (h) Fluorescence images of Se’- and Cd’-treated, and Se’-, Cd’- and GSH-treated MDCK cells 6 h after the addition of CdCl_2_. The insets show the line profiles of the fluorescence intensity along the dashed lines showed in the images. All imaging data (b, e–g) represent means ± SD of triplicate measurements in multiple cells. *n* = 20 for (e) and (g) cells. Se’ stands for Na_2_SeO_3_; Cd’ stands for CdCl_2_.

As we previously reported, 0.5 mM Na_2_SeO_3_ was added to the culture medium of Madin-Darby canine kidney (MDCK) cells, followed by the addition of 0.2 mM CdCl_2_ and GSH at different concentrations after 6 h (Fig. 1a) [4,9]. Subsequently, the changes in the fluorescence intensity and distribution of the treated cells over time were examined by confocal microscopy (Fig. 1b). The fluorescence intensity of non-GSH-treated cells was low and increased slightly over time. In contrast, as the treatment of GSH increases, the fluorescence intensity throughout the cell increases over time and is significantly enhanced in specific areas within the cell, showing a nucleus-like localization and morphology. The ultraviolet irradiation further confirmed that the fluorescence intensity of GSH-treated cells was significantly enhanced (Fig. S1 in the Supplementary Data online). These specific regions were confirmed to be cell nuclei by the colocalization of nucleus dye (Hoechst 33342) (Fig. 1c) and the strong fluorescence of extracted nucleus from the treated cells (Fig. 1d). These findings suggest that through our spatiotemporal coupling strategy and modulation of GSH addition, the fluorescence can be effectively enhanced throughout the cell, particularly in the nucleus.

As the concentration of GSH increased, the fluorescence intensity increased dramatically in the cell nucleus and then plateaued after ∼6 h (Fig. 1e). The fluorescence reached a maximum when the concentration of GSH reached 0.5 mM. Further increasing the concentration of GSH did not enhance the fluorescence intensity in the nucleus, but rather made it slightly decrease (Fig. 1e). The fluorescence intensity in cytoplasm showed a similar upward trend, with the maximum only ∼20% of that in the nucleus (Fig. 1f). Heat maps depict the trends of the changes in fluorescence intensity in the cell nucleus and cytoplasm (Fig. 1g). The line profile analysis of the cells treated with 0.5 mM GSH for 6 h shows that the fluorescence intensity in the nucleus was about five times stronger than that in the cytoplasm; however no significant difference was shown in the non-GSH-treated cells (Fig. 1h). Collectively, these results illustrate that the addition of GSH to Na_2_SeO_3_- and CdCl_2_-treated cells significantly enhances the fluorescence intensity in the nucleus. Based on these results, 0.5 mM GSH and a 6 h incubation time were chosen as the optimal conditions for experiments.

### Synthesis of QDs in the cell nucleus in the presence of GSH

**Figure 2.**
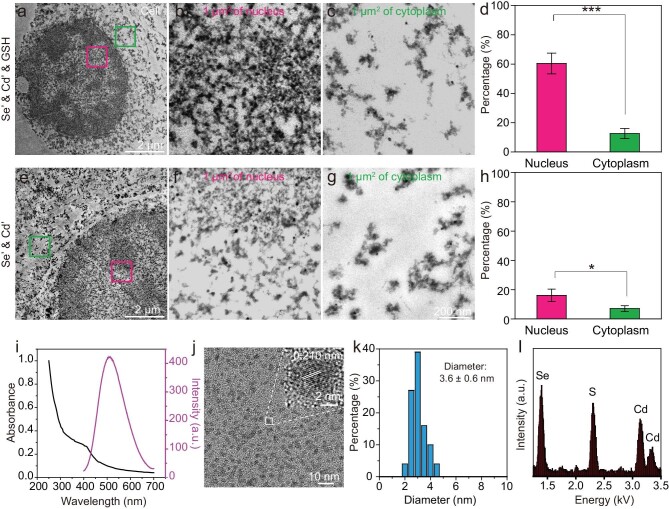
The distribution and characterization of biosynthesized QDs in MDCK cells. (a, e) TEM images of ultrathin sections of GSH-treated and untreated cells. (b, c) Magnified TEM images of the areas indicated by boxes in (a) showing 1 µm^2^ of cell nucleus and cytoplasm. (d) Percentage of the area occupied by nanoparticles in Se’-, Cd’- and GSH-treated cells. (f, g) Magnified TEM images of the areas indicated by boxes in (e) showing 1 µm^2^ of cell nucleus and cytoplasm. (h) Percentage of the area occupied by nanoparticles in Se’ and Cd’ cells. (i) Absorption and fluorescence emission of purified biosynthesized QDs. (j) High-resolution TEM image of biosynthesized QDs. (k) Size distribution of extracted biosynthesized QDs. (l) Energy dispersive spectroscopy (EDS) of biosynthesized QDs. TEM imaging data represent means ± SD of triplicate measurements in multiple cells (*n* = 20 for (d) and (h)). Statistical significance was calculated with Student's *t* test. *P < 0.05, **P < 0.01, ***P < 0.001. Se’ stands for Na_2_SeO_3_; Cd’ stands for CdCl_2_.

To clarify whether the fluorescence intensity in the nucleus was related to the QDs, ultrathin section transmission electron microscopy (TEM) was used to perform an ultrastructural analysis of the cells. The TEM results showed that both the nucleus and cytoplasm contained a large number of dark nanoparticles (Fig. 2a–c, e–g). Quantification of the area occupied by nanoparticles in the GSH-treated cells showed that the ratio of nanoparticles in the nucleus (∼60%) to the cytoplasm (∼12%) was around 5 (Fig. 2a–d), while in non-GSH-treated cells the ratio of nanoparticles in the nucleus (∼16%) to the cytoplasm (∼7%) was around 2 (Fig. 2e–h), both of which are similar to the fluorescence data shown in Fig. 1.

The nanoparticles were further extracted and purified for *in*-*vitro* analysis. The photoluminescence spectrum of the isolated nanoparticles showed an emission peak at 508 nm (Fig. 2i), consistent with the fluorescing cells (Fig. S2) [12]. High-resolution transmission electron microscopy (HRTEM) images suggested that the isolated nanoparticles were well dispersed, with an average size of 3.6 ± 0.6 nm, and the crystal-lattice distance of the nanoparticles (0.210 nm) was between those of CdSe (JCPDS No. 08-0459; 0.207 nm) and CdS (JCPDS No. 41-1049; 0.213 nm; Fig. 2j–k,), consistent with the nanoparticles synthesized in the nucleus revealed by *in-situ* HRTEM (Fig. S3). X-ray energy dispersive spectroscopy (EDS) revealed that the nanoparticles were rich in Se, S and Cd (Fig. 2l). Moreover, similar phenomena could also be observed in MCF-7 cells and HeLa cells under the same treatment (Fig. S4). Overall, these results demonstrate a GSH-facilitated *in-situ* formation of fluorescent CdS*_x_*Se_1−_*_x_* QDs in the nucleus of a wide range of cell lines.

### Unique role of GSH during the synthesis of QDs in the nucleus

In live-cell biosynthesis of QDs, GSH was found not only to act as a reducing agent, but also to be related to the synthesis location of QDs. To prove that GSH plays a pivotal role, cells were treated with other thiol-containing reducing agents such as cysteine (Cys) and dithiothreitol (DTT). Results show that Cys and DTT had little effect on the fluorescence intensity [13,14], implying that GSH plays a unique role in the biosynthesis of QDs in the nucleus (Fig. S5).

**Figure 3.**
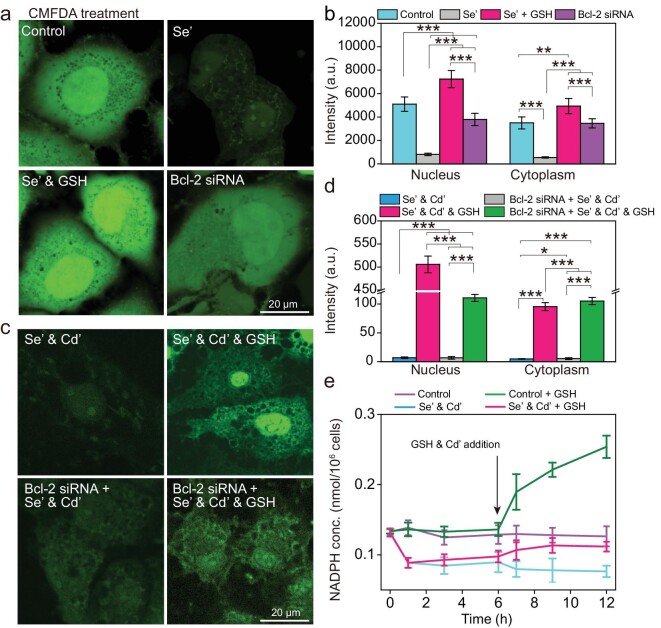
GSH plays a unique role in the synthesis of QDs in the nucleus. (a) Confocal images of control, Se’-treated, Se’- and GSH-treated, and Bcl-2 siRNA knockdown cells stained with GSH-specific dye, 5-chloromethyl fluorescein diacetate (CMFDA). (b) Quantification of the fluorescence intensity of CMFDA in the nucleus and cytoplasm of control cells, Se’-treated cells, Se’- and GSH-treated cells, and Bcl-2 siRNA knockdown cells from (a). (c) Fluorescence images of wild type (WT) and Bcl-2 knockdown cells treated with Se’ and Cd’, and Se’ and Cd’ and GSH. (d) Quantification of the fluorescence intensity in the cell nucleus and cytoplasm from (c). (e) The concentration changes of nicotinamide adenine dinucleotide phosphate (NADPH) in control cells and treated cells during QD biosynthesis. Imaging data represent means ± SD of triplicate measurements in multiple cells (*n* = 20 for (b), (d) and (e)). Statistical significance was calculated with Student's *t* test. *P < 0.05, **P < 0.01, ***P < 0.001. Se’ stands for Na_2_SeO_3_; Cd’ stands for CdCl_2_.

To explore the role and the consumption of GSH during the biosynthesis process, a GSH-specific dye, 5-chloromethyl fluorescein diacetate (CMFDA), was applied [15,16]. Images of MDCK cells stained with CMFDA show that GSH is a ubiquitous reductant in the cell and mainly accumulates in the nucleus (Fig. 3a). Quantification of CMFDA fluorescence revealed that the concentration of GSH in the nucleus was ∼1.5 times higher than that in the cytoplasm (Fig. 3b). After Na_2_SeO_3_ treatment, the CMFDA fluorescence of the whole cell decreased significantly by 80%, indicating that the GSH was greatly consumed during the selenization process, which was further confirmed through quantification of total cellular GSH (Fig. S6). These results suggest that GSH participates in the biosynthesis of QDs as a reducing agent for the formation of Se precursors, and selenization mainly occurs in the nucleus owing to the uneven distribution of GSH. With the addition of GSH, the fluorescence intensity of CMFDA increased significantly throughout the cell, especially in the nucleus, indicating that the supplementation of GSH compensates the consumed GSH during selenization and induces the enrichment of the re-supplemented GSH in the nucleus.

In addition, Bcl-2 has been reported as a GSH transporter as it assists GSH in translocating from the cytoplasm to the nucleus [17–20]. To test this notion, MDCK cells were pretreated with Bcl-2-targeted small interfering RNA (siRNA) to efficiently suppress Bcl-2 expression (Fig. S7). The CMFDA treatment of Bcl-2 knockdown cells showed a similar distribution of fluorescence across the cell, demonstrating that Bcl-2 is responsible for transporting GSH from the cytoplasm to the nucleus and is the main cause of this uneven distribution (Fig. 3a, b). Consistently, the fluorescence intensity of biosynthesized QDs in the nucleus and cytoplasm of Bcl-2 knockdown cells was almost equal (Fig. 3c, d). When the Bcl-2 knockdown cells were treated with GSH, the fluorescence intensity in the nucleus also approached that in the cytoplasm, which was ∼80% lower than that in the nucleus of wild-type cells (Fig. 3c, d). Overall, these results indicate that Bcl-2 is responsible for transporting the GSH into the nucleus to form a GSH pool, which participates in the selenization reaction in the nucleus, achieving the *in-situ* synthesis of QDs.

### Enrichment of glutathione reductase in the cell nucleus during the synthesis of QDs in the nucleus

The biosynthesis of QDs includes two phases: cell selenization, and Cd^2+^ binding and crystallization [21]. Selenite is commonly thought to be reduced to selenodiglutathione (GSSeSG) by GSH and further reduced to low-valence Se (e.g. GSSeH and HSe^−^) by GSH-related enzymes such as glutathione reductase (GR) and nicotinamide adenine dinucleotide phosphate (NADPH) [4,6,7,22–24]. The high-performance liquid chromatography coupled with inductively coupled plasma mass spectrometry (HPLC-ICP-MS) analysis confirmed that our approach can produce low-valence Se compounds, including selenocystine (SeCys) and selenomethionine (Se-MC; Fig. S8). Next, we identified the crucial biomolecules related to the biosynthesis of QDs.

**Figure 4.**
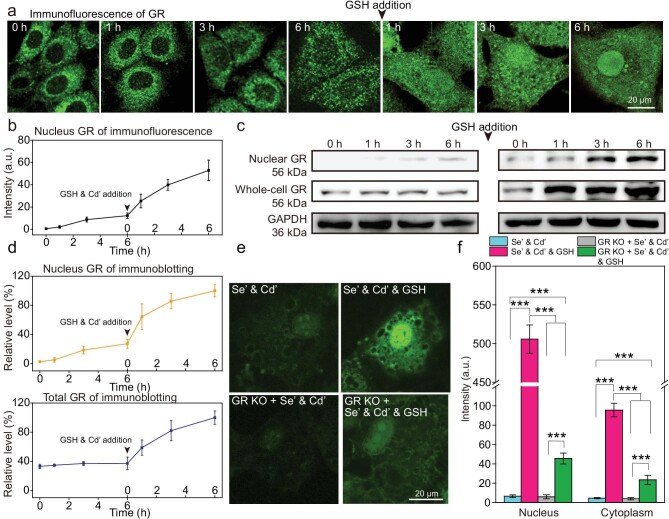
Enrichment of GR in the cell nucleus during QD synthesis. (a) Time-dependent immunofluorescence images of GR during the QD biosynthesis in MDCK cells. (b) Quantification of the immunofluorescence intensity of GR in the cell nucleus at different time points from (a). (c) Immunoblotting of GR in the nucleus and whole cell during QD biosynthesis. Representative Western blots (*n* = 3) show GR detected by immunoblot assay with the GR-specific antibody. Glyceraldehyde 3-phosphate dehydrogenase (GAPDH) was used as a loading control. Time was calculated from the addition of Se’ and restarted when GSH and Cd’ were added. (d) Quantitative densitometry analysis of GR in the nucleus and whole cell bands shown in (c). (e) Confocal images of QD signals in WT cells and GR-knockout (KO) cells treated with Se’ and Cd’, or Se’ and Cd’ and GSH. (f) Quantification of the fluorescence intensity of QDs in the nucleus and cytoplasm of the WT and GR-KO cells treated with Se’ and Cd’, or Se’ and Cd’ and GSH. Imaging data represent means ± SD of triplicate measurements in multiple cells (*n* = 20 for (b and f)). Statistical significance was calculated with Student's *t* test. *P < 0.05, **P < 0.01, ***P < 0.001. Se’ stands for Na_2_SeO_3_; Cd’ stands for CdCl_2_.

The spatial distribution of GR within the cells during selenization was examined by immunofluorescence staining as it recycles GSH from glutathione disulfide (GSSG). Images showed that during selenization, GR was gradually enriched in the nucleus and could be clearly observed after 3 h (Fig. 4a, b). Furthermore, the Western blotting data showed that the total cell GR content increased slightly after selenization for 6 h, but the nuclear GR content increased significantly (Fig. 4c, d). These results imply that GR enrichment is mainly due to translocation of GR from the cytoplasm to the nucleus during the selenization process. After GSH treatment, GR expression was significantly up-regulated in the whole cell and further enriched in the nucleus, indicating the importance of GR in nuclear QD biosynthesis (Fig. 4c, d, and Fig. S9). Finally, whether GR expression impacts the formation of QDs in the nucleus was investigated. When GR expression was suppressed by siRNA (Fig. S10a), the fluorescence intensity in the nucleus and cytoplasm decreased dramatically. The addition of GSH only slightly recovered the QD fluorescence of whole cells (Fig. S10b, c). Similar results were obtained in MDCK cells with GR knockout using CRISPR-Cas9 technology, suggesting that GR is largely involved in the formation of QDs (Fig. S11, and Fig. 4e, f) [25].

Given that NADPH is an electron donor for the GR-catalyzed reduction of GSSG to GSH, the consumption of NADPH during the biosynthesis of QDs was examined [26]. The quantification of NADPH showed a steep decrease in its concentration with the addition of Na_2_SeO_3_, indicating that NADPH participated in the selenization process. With the addition of GSH and Cd^2+^, the concentration of NADPH increased slightly in Na_2_SeO_3_-treated cells and significantly in normal cells, suggesting that the addition of GSH may up-regulate the intracellular level of NADPH, which was rapidly consumed during QD biosynthesis (Fig. 3e). Further, the treatment of diphenylene iodonium (DPI, an inhibitor for NADPH synthesis) almost completely blocked the ability to synthesize QDs in the cell (Fig. S12) [27]. Consequently, the NADPH was presumably involved in the reduction of Se and essential for the generation of QDs.

### Formation of cadmium precursors during the synthesis of QDs in the nucleus

For the formation of cadmium precursors, many thiol- or selenol-containing biomolecules (RSH) may interact with Cd^2+^, which can be transported into the cell through Ca^2+^-related transport proteins [28], to promote Cd^2+^ sequestration. Time-dependent changes in RSH concentration showed that the RSH concentration was significantly improved by GSH addition (Fig. S13), indicating that RSH may be involved in QD biosynthesis. This was further verified by the severely suppressed QD biosynthesis after adding *N*-ethylmaleimide (NEM, an RSH inhibitor) (Fig. S14) [29,30]. The question that arises is how RSH is involved in the biosynthesis of QDs. Thus, solutions have been attempted by simulating the binding model of various Cd precursors.

**Figure 5.**
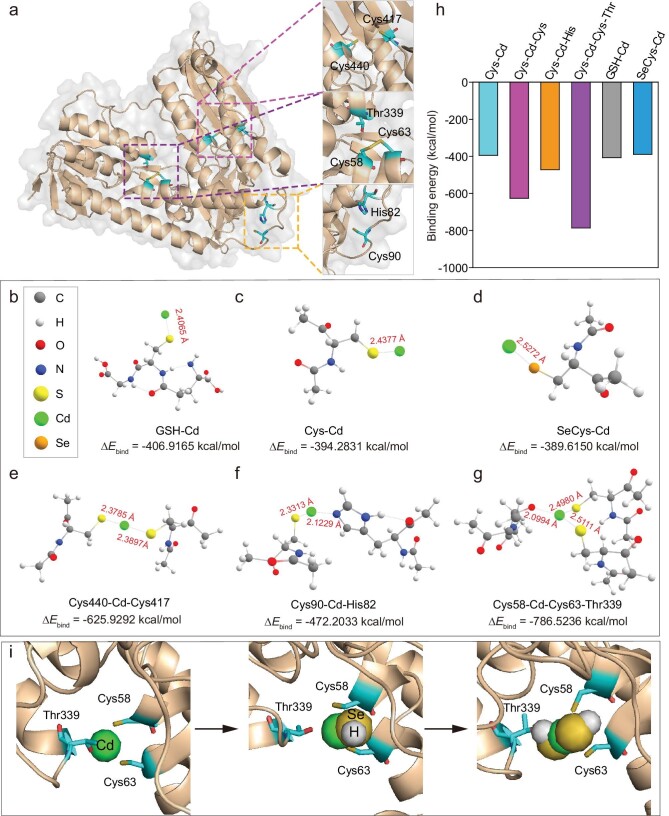
Density functional theory (DFT) calculations and molecular dynamic (MD) simulation reveal the interactions of GR with Cd^2+^ as well as the formation of the Cd precursor. (a) The crystal structure of GR with enlarged possible Cd^2+^ binding sites. (b–g) DFT optimized structures of GSH-Cd, Cys-Cd, SeCys-Cd, Cys-Cd-Cys, Cys-Cd-His and Cys-Cd-Cys-Thr. (h) The binding energies for the six models shown in (b–g). (i) Interactions between Cys58, Cys63, Thr339 and Cd^2+^ with supply of one HSe^−^ and two HSe^−^. Initially, the Cys-Cd-Cys-Thr, consisting of Cd^2+^ coordinated with two S atoms and one O atom, was treated as the initial nucleus of QDs. HSe^−^ added at a distance of 3.5 Å from Cd^2+^ could coordinate with Cd^2+^, accompanied by the separation of O (Thr339) and Cd^2+^. Another HSe^−^ added at a distance of 3.5 Å from Cd^2+^ could also coordinate with Cd^2+^ and lead to the distance changes between O (Thr339) and Cd^2+^.

First, GR (Protein Data Bank ID: 3DK4) was chosen as a cysteine-rich protein model to explore the molecular mechanism of Cd precursor generation, since GR is mainly involved in the biosynthesis of QDs (Fig. 5a). We first optimized binding modes of free GSH, cysteine and SeCys to Cd^2+^ using density functional theory (DFT). Similar binding forces were found between Cd^2+^ and these three molecules, but were relatively strong with GSH. Considering that multiple thiols and selenols are usually exposed in the protein structure, they could also be potential binding sites for Cd^2+^ (Fig. 5b, d, h). Then the structures of the possible binding sites on the GR were optimized. The two Cys sites (Cys440, Cys417) of GR have higher Cd^2+^ affinity than the single cysteine, indicating that more ligands reduce the binding energy (Δ*E*_bind_) and contribute to the formation of the coordination compound. Also, considering that other groups of amino acids (e.g. imidazole of histidine and hydroxyl of threonine) may also interact with Cd^2+^, the binding energies of Cys-Cd-Cys (Cys440, Cys417) with Cys-Cd-His (Cys90, His82) were compared, showing that the Cys-Cd-Cys had a much lower Δ*E*_bind_ (−625.9292 kcal/mol) than Cys-Cd-His (−472.2033 kcal/mol), indicating that the Cd^2+^ prefers to bind to thiol compared with imidazole. Meanwhile, the Cys-Cd-Cys-Thr (Cys58, Cys63, Thr339) model displayed a stronger energy (Δ*E*_bind_: −786.5236 kcal/mol) than Cys-Cd-Cys, implying that the interaction between Cd^2+^ and hydroxyl of threonine can further increase the RSH's ability to sequester Cd^2+^. Taken together, the Cd^2+^ had the strongest binding energy (Δ*E*_bind_: −786.5236 kcal/mol) in the Cys-Cd-Cys-Thr model and the weakest Δ*E*_bind_ (−394.2831 kcal/mol) in the Cys-Cd model, suggesting that more ligands reduce the binding energy and promote Cd^2+^ sequestration (Fig. 5e, h, Table S1).

According to the reports, selenium compounds (e.g. GSSeSG and GSSeH) can be metabolized by GR, NADPH and GSH to hydrogen selenide (HSe^−^), but HSe^−^ is too reactive to be directly identified [24,25]. The nucleation process of QDs was investigated by molecular dynamic (MD) simulation of the interaction process between the sequestered Cd^2+^ and HSe^−^. Here, the Cys-Cd-Cys-Thr, consisting of Cd^2+^ coordinated with two S atoms and one O atom (each from Cys58, Cys63 and Thr339), was treated as the initial nucleus of QDs. Firstly, the distances between O (Thr339), S (Cys58) and S (Cys63) and Cd^2+^ were statistically analyzed, and the results demonstrate that these distances remained constant within 10 ns, indicating that all three coordination atoms can be stably bound to Cd^2+^ (Fig. 5i and Fig. S15a). In this equilibrated structure, HSe^−^ was added at a distance of 3.5 Å from Cd^2+^ and could coordinate with Cd^2+^ instantaneously (<0.1 ns) and remained stable for 380 ns, accompanied by the separation of O (Thr339) and Cd^2+^, indicating a strong affinity of HSe^−^ for Cd^2+^ (Fig. 5i and Fig. S15b). Based on this stable state, another HSe^−^ was added at a distance of 3.5 Å from Cd^2+^. Similarly, the HSe^−^ was quickly (<0.1 ns) coordinated with Cd^2+^, and remained stable for 380 ns. Intriguingly, in this process, O (Thr339) was again coordinated with Cd^2+^, indicating that coordination between O (Thr339) and Cd^2+^ occurs randomly due to weak interactions, and O (Thr339) may be replaced by stable coordination when more HSe^−^ interacts with Cd^2+^(Fig. 5i and Fig. S15c). In this pattern, the QD grows gradually with the continuous supplement of Cd^2+^ and HSe^−^, and eventually forms CdS*_x_*Se_1−_*_x_* QD. The simulation results revealed no significant alterations in the protein's structural framework following the introduction of Cd^2+^ (Fig. S16).

## DISCUSSION

**Figure 6.**
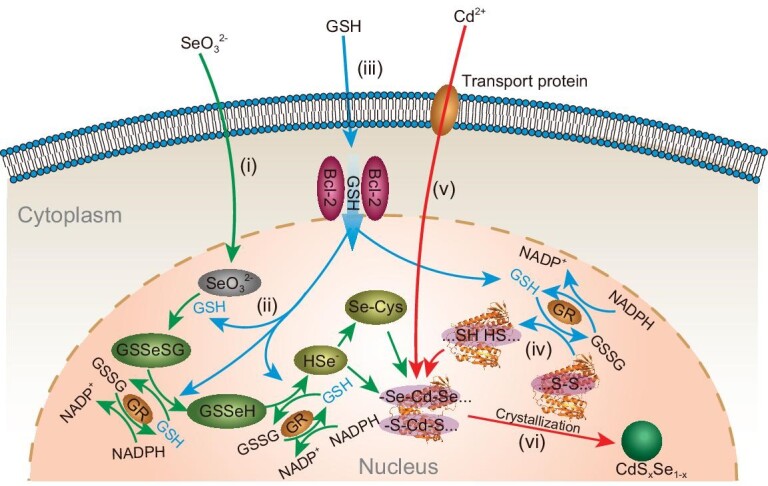
A scheme for the biosynthesis of QDs in live cells. (i, ii) After Na_2_SeO_3_ is added to the cell culture medium, SeO_3_^2−^ can permeate into the cell nucleus, which is then reduced to GSSeSG by GSH, and further reduced to low-valence Se, such as HSe^−^ or SeCys, through the co-effect of GSH, GR and NADPH. This process induces the enrichment of GR inside the cell nucleus. (iii) After 6 h of selenization, the extra GSH is added to the cell, mostly via Bcl-2 transport to the nucleus, which can supplement the reducing power to facilitate step (ii), or promote RSH production on cellular proteins (iv). (v) Cd^2+^ can be transported into the cell by Ca^2+^-related transport proteins and penetrate into the nucleus, and is readily sequestered by RSH, especially with multiple adjacent thiols, selenols, hydroxyl groups and imidazoles to form Cd precursors. (vi) As the initial nucleus of QD, the Cd precursor gradually crystallizes over time and grows in size, eventually forming CdS*_x_*Se_1−_*_x_* QDs.

Although the synthesis of QDs in different cell lines has been extensively studied, questions regarding biosynthesis in organelles in live cells, particularly the nucleus, remain unanswered. Here, we achieved the *in-situ* synthesis of CdS*_x_*Se_1−_*_x_* QDs in the live nucleus for the first time by modulating the GSH metabolic pathway, a conceptual breakthrough in the biosynthesis of nanomaterials. With the disproportionate distribution of GSH in live cells, we enabled the *in-situ* synthesis of QDs in the nucleus of live cells and revealed the mechanism of intranuclear synthesis of QDs associated with GSH and GR (Fig. 6). Our results reveal that, after the addition of Na_2_SeO_3_ in cell culture medium, SeO_3_^2−^ can permeate into the cell nucleus, which is then reduced to GSSeSG by GSH, and further reduced to low-valence Se, such as HSe^−^ or SeCys through the co-effect of GSH, GR and NADPH (Fig. 6i–ii). Increased GSSeSG levels in the nucleus lead to the selenization process accompanied by GR enrichment in the nucleus. Extra GSH is added to the cell and is translocated to the nucleus primarily via Bcl-2 (Fig. 6iii), which can further complement the reducing power to promote RSH production on cellular proteins (Fig. 6iv). Then, Cd^2+^ can be transported into the cell by Ca^2+^-related transport proteins and penetrates into the nucleus, and is readily sequestered by RSH, especially with multiple adjacent thiols, selenols, hydroxyl groups and imidazoles to form Cd precursors (Fig. 6v). As the initial nucleus of QD, the Cd precursor gradually crystallizes over time and grows in size, eventually forming CdS*_x_*Se_1−_*_x_* QDs (Fig. 6vi). These findings present a conceptual innovation in the intracellular synthesis of QDs in live cells and complements organelle-oriented biosynthesis of QDs, particularly in the nucleus.

Previous studies usually focused on the synthesis of QDs in different organisms, while the targeted synthesis of QDs at the organelle level has been relatively constrained. The *in-situ* synthesis of CdS*_x_*Se_1−_*_x_* QDs within the cell nucleus has demonstrated the remarkable potential of cellular systems for nanomaterial synthesis, and the ability to synthesize QDs specifically in cellular organelles, providing new insights into the field of biosynthesis. Furthermore, previous studies have shown that different types of QDs (e.g. CdS, CdTe and ZnSe) can be successfully synthesized in different organisms [31–33]. Most of these studies utilized GSH as an important reducing agent. This feasibility study has unveiled the possibility of expanding the range of QD types that can be synthesized within the cell nucleus, and generating these types of QDs in the nucleus of mammalian cells by monitoring GSH levels. Therefore, our work provides key insights into the mechanism of the synthesis of exogenous substances in the nucleus, contributing to a new understanding of the synthetic function of the nucleus.

Despite the promising prospects, certain challenges and issues remain to be addressed. Firstly, although biosynthesized QDs are biocompatible, the precursors (e.g. Na_2_SeO_3_ and CdCl_2_) required may be relatively harmful to cells. Addressing the toxicity of the QDs is imperative to expanding the application of this method to intracellular applications, and the synthesis of functional nanomaterials free of toxic metals would be very valuable. Secondly, the mechanism proposed in this paper realizes QD synthesis exclusively in the nucleus, and there are a lack of attempts to synthesize QDs *in situ* in other organelles. Therefore, developing methods for the specific *in-situ* synthesis of QDs in other organelles is still needed. Lastly, the biosynthesized QDs exhibit lower brightness than chemically synthesized QDs. Increasing the quantum yield by structural improvement or exploring the synthesis of core-shell alloy QDs may be crucial to enhancing their brightness. Taken together, these findings provide a potential pathway for intracellularly oriented synthesis, artificially realizing processes that occur non-normally in the nucleus. This advances the understanding and development of the artificially regulated live-cell synthesis of QDs, and continued progress will naturally lead to significant and meaningful discoveries and applications in the future.

## Supplementary Material

nwae021_Supplemental_File

## METHODS

Methods are available in the Supplementary Data at *NSR* online.
