# Supplementary material for: In-situ synthesis of quantum dots in the nucleus of live cells
Source: Natl Sci Rev. 2024 Jan 12;11(3):nwae021. doi: 10.1093/nsr/nwae021 (PMC10896589; doi:10.1093/nsr/nwae021)
Supplement: nwae021_Supplemental_File [file nwae021_supplemental_file.doc]

**SUPPLEMENTARY INFORMATION**

***In-situ* synthesis of quantum dots in the nucleus of live cells**

Yusi Hu^1,2,#^, Zhi-Gang Wang^1,2,#^, Haohao Fu^1,2^, Chuanzheng Zhou^1^, Wensheng Cai^1,2^, Xueguang Shao^1,2^, Shu-Lin Liu^1,2^, Dai-Wen Pang^1,2^,*

^1^State Key Laboratory of Medicinal Chemical Biology, Frontiers Science Centre for New Organic Matter, Tianjin Key Laboratory of Biosensing and Molecular Recognition, Research Centre for Analytical Sciences, College of Chemistry, School of Medicine, and Frontiers Science Centre for Cell Responses, Nankai University, Tianjin 300071, P. R. China.

^2^Haihe Laboratory of Sustainable Chemical Transformations, Tianjin 300192, P. R. China.

^#^These two authors contribute equally to this work.

*Email: [dwpang@whu.edu.cn](mailto:dwpang@whu.edu.cn)

## Supplementary Material and Methods

**Cell culture.** MDCK cells, HeLa cells, and MCF-7 cells were maintained in culture medium (Dulbecco’s modified Eagle medium [DMEM]; Gibco) with 10% fetal bovine serum (FBS; Gibco), 100 U/mL penicillin G, and 100 µg/mL streptomycin sulfate at 37 °C in a 5% CO_2_ incubator and passaged every 2–3 days.

**Quantum dots characterization.** The fluorescence spectrum and lifetime of extracted QDs or fluorescence cells were characterized by fluorescence spectrometer (Edinburgh Instruments, FLS1000), the absorbance of quantum dots was measured by UV-VIS-NIR spectrophotometer (Shimadzu, UV-3600 Plus). The morphologies, crystal-lattice distance and EDS were characterized by transmission electron microscopy (FEI, Taols F200X G2).

**Biosynthesis of QDs in mammalian cells.** Cells were grown as monolayers in DMEM containing 10% FBS, 100 U/mL penicillin G sodium, and 100 µg/mL streptomycin sulfate at 37 °C in a 5% CO_2_ atmosphere to ensure the cells were maintained in the stationary phase. Cells were seeded in 100 mm plates 36 h before being incubated with Na_2_SeO_3_ (final concentration: 0.5 mM) for 6 h in the same DMEM as above with 2% FBS. After treatment, the medium was changed, and CdCl_2_ (final concentration: 0.2 mM) and GSH (final concentration: 0.5 mM) were added to medium. Cells were harvested or imaged after 6 h of incubation if not otherwise specified.

**Extraction of biosynthesized QDs.** The cells were collected and centrifuged at 1000 ×g for 5 min. The cell pellet was washed and resuspended in phosphate buffered saline (PBS). The cells were then sonicated for 10 min, with 2 s working time and a 2 s interval (Scientz, JY96-IIN). The suspension was centrifuged at 12,000 ×g for 15 min to precipitate out the cell fragments. Then the CdSe QDs were purified by filtration with a centrifugal filter (Amicon Ultra-4 30k, Millipore, USA) to further remove the compounds less than 30 kDa.

**Extraction of the cell nucleus.** The cell nucleus was extracted with an extraction kit (Solarbio Cat. No. SN0020).

**siRNA knockdown.** MDCK cells were plated on 6-well plates with around 1.5 × 10^5^ cells and incubated for 24 h. Three siRNA solutions, each 10 pmol, were transfected into cells with jetPRIME (Polyplus-transfection) according to the manufacturer’s protocol. After 48 h of transfection, the cells were harvested for Western blotting. siRNA strands were purchased from Sangon Biotech.

**Western blotting.** Transfected cells or cell nuclei were lysed in cell lysis buffer (Solarbio Cat. No. BC3710). The protein density was quantified by Bradford assay (Solarbio Cat. No. PC0010). The equivalent amount of protein was loaded onto polyacrylamide gel to run sodium dodecylsulfate polyacrylamide gel electrophoresis (SDS-PAGE). Proteins were separated and transferred to a polyvinylidene difluoride membrane (Immobilon®-P^SQ^ Membrane, Cat. No. ISEQ00010). The membrane was blocked with 5% (w/v) BSA (Solarbio Cat. No. A8010) for 45 min and incubated overnight at 4 °C with primary antibodies (1:2000 dilution). After the unbound antibodies were removed, the membrane was incubated with secondary antibodies (1:2000 dilution) for 1 h at room temperature. Images were taken with a BioRad Chemidoc™ MP imaging system. The antibodies used included Bcl-2 (Solarbio Cat. No. K200018M), GR (Solarbio Cat. No. K002418P), and GAPDH (SinoBiological Cat. No. 10094-T52).

**Quantitative Real-Time PCR.** Total RNAs were extracted from cells by the Universal RNA Extraction Kit (Takara, Cat. No. 9767). The concentration and purity of the final RNA were determined through Multiscan Sky (ThermoFisher Scientific Inc., U.S.A.). The cDNA was synthesized using the PrimeScript 1^st^ Strand cDNA Synthesis Kit (Takara, Cat. No. 6110A). The qRT-PCR analysis was performed using SYBR Premix (GenStar, Cat. No. A304-10), running on the QuantStudio 3 real-time PCR system (ThermoFisherScientific Inc., U.S.A.). For each sample, the average of the three replicates was used to calculate transcript abundance. For normalization, GAPDH was used as the internal standard. The relative cDNA abundance of each gene in the control was set as 1.0.

**Quantification and statistical analysis.** The fluorescence imaging data and line profile were analyzed with Image Pro-Plus (IPP). The band intensities of the Western blotting were quantified with ImageJ (Bethesda, MD, USA). Statistical significance was calculated with Student’s t test. Significance was set at *p < 0.05, **p < 0.01, ***p < 0.001. The number of experiments and the total number of cells analyzed (*n*) are reported in the Figure legends.

**CMFDA staining.** CMFDA was purchased from Aladdin (Cat. No. C131098). Cells were washed with PBS, then treated with 2 µM CMFDA, avoiding light, at 37 °C for 1 h.

**Hoechst 33342 staining.** Hoechst 33342 staining solution was purchased from Sangon Biotech (Cat. No. E607302). Cells were washed with PBS three times, then incubated with Hoechst 33342 in PBS at a 1:100 dilution at 37 °C for 30 min. Cells were then washed with PBS three times and imaged.

**Measurement of total GSH.** A Reduced GSH Assay Kit was purchased from Solarbio (Cat. No. BC1170). Cells were treated with Na_2_SeO_3_ for specified numbers of hours, collected, and counted with a cell counter (Countess II FL Automated Cell Counter, AMQAF1000, Thermo Fisher Scientific, USA). The amount of GSH was measured with the assay kit following the manufacturer’s protocol.

**Measurement of total NADPH.** A CheKine™ Coenzyme II NADP(H) Assay Kit was purchased from Abbkine (Cat. No. KTB1010). Cells were treated with Na_2_SeO_3_ for specified numbers of hours, collected, and counted with a cell counter (Countess II FL Automated Cell Counter). The amount of NADPH was measured with the assay kit following the manufacturer’s protocol.

**Characterization of intracellular reduced Se species.** After treatment with Na_2_SeO_3_, the cell nucleus was extracted as mentioned previously. Briefly, 0.5 g wet weight cell nucleus was harvested and washed once with PB buffer (pH 7.2, 0.01 M) and ultrasonicated at 200 W, 25 times a cycle, 5 s per time, 2 s intervals each time. The lysed cells were centrifuged at 12,000 rpm for 30 min, the supernatant was collected, and the procedure was repeated. Trypsin from bovine pancreas (final concentration: 250 µg/mL; Sigma) and 0.1% (w/v) SDS were added to the supernatant for digestion for 24 h at 37 °C. The supernatant was filtered through a 0.22 µm filter, and a 100 µL aliquot was injected for HPLC measurement. Sample separation was performed on an LC-10AD HPLC system (Shimadzu, Japan) equipped with a Superdex 200 10/300 GL gel filtration column (13 µm, 10 × 300 mm; GE Healthcare, Amersham Biosciences). The separation fraction (82.5–90 min) was collected and injected into HPLC-ICP-MS for further analysis. Sample separation was performed on an ultimate 3000 HPLC system (Dionex, USA) and a C18 column acclaim 120 (150 × 2.1 mm I.D., 3 µm, Dionex, USA) coupled with an X series II ICP-MS (Thermo Fisher Scientific) for online element-specific detection.

**NEM treatment.** MDCK cells were treated with 100 µM NEM at 37 °C for 30 min before seleniumization as well as CdCl_2_ and GSH addition.

**RSH concentration measurement.** As previously reported [1], 10^6^ cells were collected in, washed twice through ice-cold double-distilled water (ddH2O), resuspended in 1 mL of HCl solution (pH=4.5), and then ultrasonicated (2 s with 2 s interval, 15 min). The sample (100 μL) was then treated with same volume of RSH assay buffer (pH=4.5). After 30 min incubation at 30 °C, then the reaction was quenched by the addition of HCl to a final content of 0.2 M. The reaction product was analyzed by reverse-phase high-performance liquid chromatography (HPLC) using C-18 reversed-phase column. The RSH were separated using 50 mM potassium acetate (pH=4.0) mobile phase at a flow rate 1 mL/min and were monitored through measure the 324 nm absorbance. The RSH assay buffer contains 0.72 mM 4,4’-dithiodipyridine (4-DPS), 0.2 M citrate, 0.4 mM ethylenediaminetetraacetic acid (EDTA), and 12 M urea.

**Live cell imaging.** Treated cells were imaged with a spinning-disk confocal super-resolution microscope (Olympus IXplore SpinSR10) under a 100× objective. Cells containing biosynthesized QDs were excited with a 405 nm diode pumped solid state (DPSS) laser and the emission was filtered with a 525/50 nm band-pass filter . CMFDA-stained cells were imaged with a 488 nm DPSS laser and the emission was filtered with a 525/50 nm band-pass filter.

**Immunofluorescence.** MDCK cells were treated as mentioned in the paper, and they were washed with PBS for 3 min three time. Cells were fixed with 4% (m/v) paraformaldehyde for 30 min at room temperature. Cells were washed with PBS for 3 min 3 time and incubated with 0.5% Triton X-100(v/v) for 20 min at room temperature. Cells were washed with PBS for 10 min three time and blocked with 5% (m/v) bovine serum albumin (BSA) for 30 min at room temperature. Cells were incubated with primary antibody (GR, Solarbio Cat. No. K002418P) overnight at 4 °C or for 2 h at room temperature. Cells were washed with PBS for 10 min three time and incubated with the secondary antibody (abbkine Cat No. A23620) for 45 min under dark. Cell were washed with PBS for 10 min three time and imaged using 640 nm DPSS laser and the emission was filtered with a 685/40 nm band-pass filter.

**Computational methods.** The complex structures Cys-Cd-Cys, Cys-Cd-His, and Cys-Cd-Cys-Thr were extracted from the crystal structure (PDB ID: 3DK4), among which the C-terminus and N-terminus of Cys, His, and Thr amino acids were extended with methyl and acetyl groups, respectively. Cys-Cd, SeC-Cd and GSH-Cd were also constructed.

The six complex systems were optimized and extracted and the binding energies calculated at the B3LYP[2]-D3[3]/DEF2-TZVP[4] level. The basis set superposition error (BSSE) was added during the calculation [5-6]:

**E***_bind_* = *E(complex) – E(A) – E(B) + E_BSSE_*

Here, *E(complex)* is the energy of the complex, *E(A)* is the energy of molecule A, *E(B)* is the energy of molecule B, and *E_BSSE_* represents the corrected energy of the BSSE. *E_BSSE_* can also be calculated using the following formula:

**E***_BSSE_* = [*E(A) – E_complex_(A)*] + [*E(B) – E_complex_(B)*]

Here, *E_comple_*_x_*(A)* represents the energy of molecule A under the basis function of the complex, and *E_complex_(B)* represents the energy of molecule B under the basis function of the complex. Therefore, the binding energy can be calculated with the following formula:

**E***_bind_* = *E(complex) – E_complex_(A) – E_complex_(B)*

**MD simulations.** MD simulations were performed using the NAMD software [7]. The CHARMM36m force field was used for proteins and ions [8]. The parameters of the HSe^-^ molecule were taken from reference [9]. Each system was solvated with TIP3P explicit water molecules, and counterions were added to neutralize its total charge.

Each simulation was first subjected to minimization with the protein held fixed, followed by an additional minimization with all atoms allowed to relax. Equilibration was performed at a constant temperature of 300 K and a constant pressure of 1 atm using Langevin dynamics and Langevin piston [10]. NPT simulations used the same settings for the thermostat and barostat as during equilibration. A 2.0 fs time step was utilized and the bonds involving hydrogen atoms were constrained by the SHAKE algorithm. A 12-Å cutoff was used to truncate the short range van de Waals and Coulomb interactions. Long-range Coulomb interactions were estimated using the particle-mesh-Ewald (PME) method [11].

In MD simulation, we first constrained all solute atoms at their initial positions, balanced for 10 ns, then released constraints except Thr339, Cys58, Cys63 and Cd^2+^. After balancing for another 10 ns, we finally released all constraints in the production simulations.

## Reference:

1. Hansen, R. E.; Ostergaard, H.; Norgaard, P.*, et al.*, Quantification of protein thiols and dithiols in the picomolar range using sodium borohydride and 4,4'-dithiodipyridine. *Anal. Biochem.* 2007**,** **363**, 77-82.

2. Yanai, T.; Tew, D. P.; Handy, N. C., A new hybrid exchange–correlation functional using the Coulomb-attenuating method (CAM-B3LYP). *Chem. Phys. Lett.* 2004**,** **393**, 51-57.

3. Grimme, S.; Antony, J.; Ehrlich, S.*, et al.*, A consistent and accurate ab initio parametrization of density functional dispersion correction (DFT-D) for the 94 elements H-Pu. *J. Chem. Phys.* 2010**,** **132**, 154104.

4. Rappoport, D.; Furche, F., Property-optimized gaussian basis sets for molecular response calculations. *J. Chem. Phys.* 2010**,** **133**, 134105.

5. Boys, S. F.; Bernardi, F., The calculation of small molecular interactions by the differences of separate total energies. Some procedures with reduced errors. *Mol. Phys.* 2006**,** **19**, 553-566.

6. Simon, S. l.; Duran, M., How does basis set superposition error change the potential surfaces for hydrogen-bonded dimers? *J. Chem. Phys.* 1996**,** **105**, 11024.

7. Phillips, J. C.; Hardy, D. J.; Maia, J. D. C.*, et al.*, Scalable molecular dynamics on CPU and GPU architectures with NAMD. *J. Chem. Phys.* 2020**,** **153**, 044130.

8. Huang, J.; Rauscher, S.; Nawrocki, G.*, et al.*, CHARMM36m: an improved force field for folded and intrinsically disordered proteins. *Nat. Methods* 2017**,** **14**, 71-73.

9. Tian, L. J.; Min, Y.; Li, W. W.*, et al.*, Substrate metabolism-driven assembly of high-quality CdSxSe1-x quantum dots in Escherichia coli: molecular mechanisms and bioimaging application. *ACS Nano* 2019**,** **13**, 5841-5851.

10. Feller, S. E.; Zhang, Y.; Pastor, R. W., Constant pressure molecular dynamics simulation: The Langevin piston method. *J. Chem. Phys.* 1995**,** **103**, 4613-4621.

11. York, D. M.; Darden, T. A.; Pedersen, L. G., The effect of long-range electrostatic interactions in simulations of macromolecular crystals: A comparison of the Ewald and truncated list methods. *J. Chem. Phys.* 1993**,** **99**, 8345-8348.

## Supplementary Figures and Table


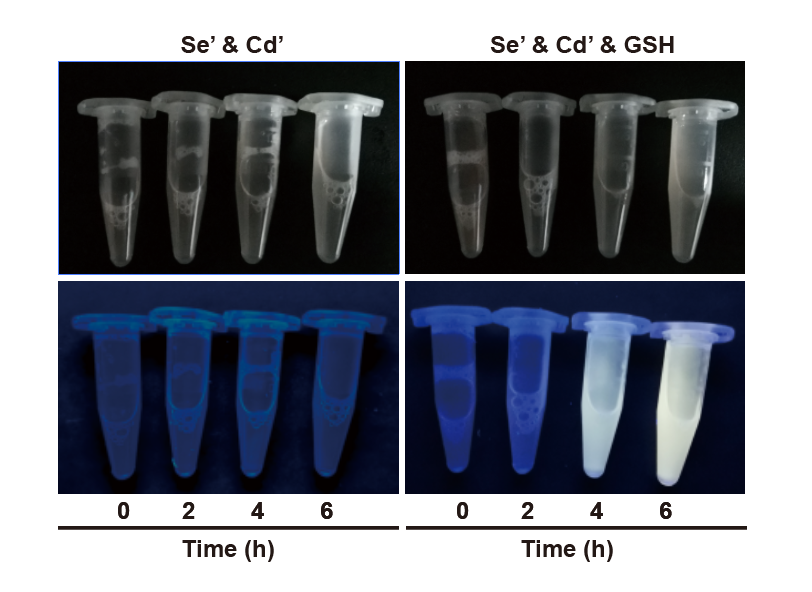


**Figure S1:** The effect of GSH on the QD production in cells incubated with Na_2_SeO_3_ and CdCl_2_. Images are of treated cells over synthesis time under bright-field (top) and ultraviolet irradiation (bottom). Se’ stands for Na_2_SeO_3_, Cd’ stands for CdCl_2_.

**
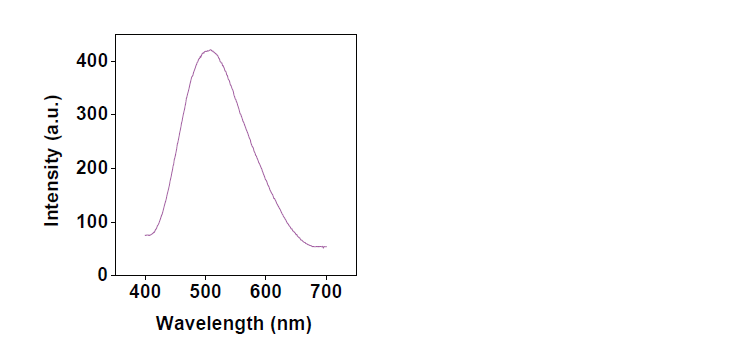
**

**Figure S2:** Fluorescence spectra of fluorescent cells.


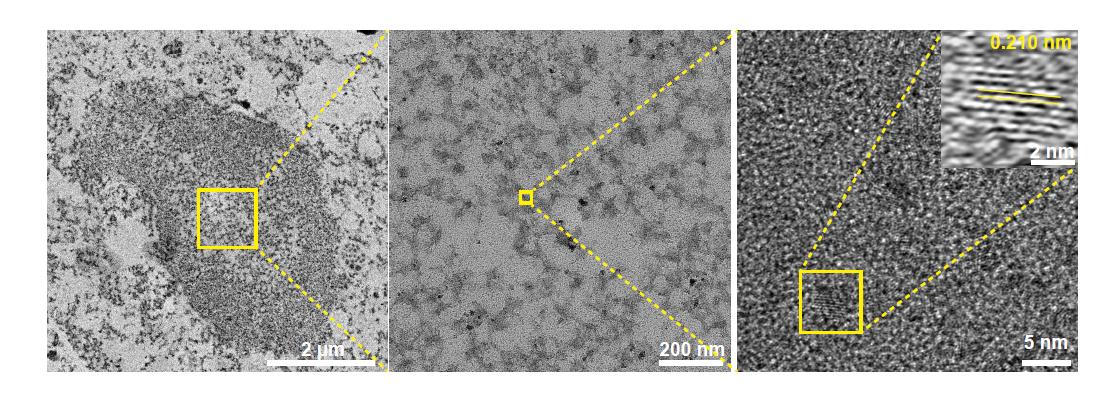


**Figure S3**: *In-situ* HRTEM images of intracellular QDs revealing the nanoparticle as a single crystalline with a lattice spacing of approximately 0.210 nm.


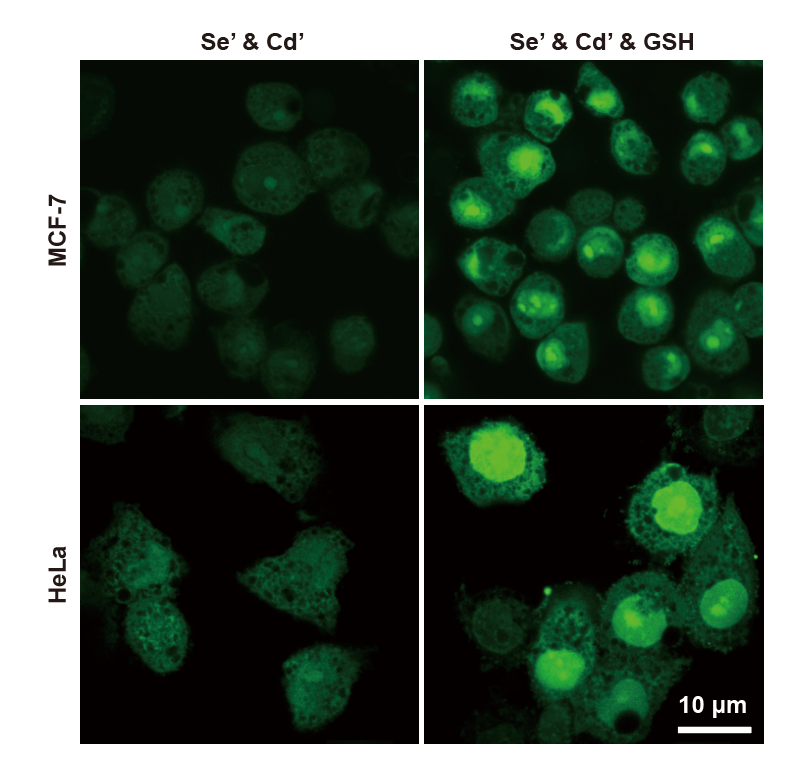


**Figure S4**: Fluorescence images of MCF-7 and HeLa cells treated with Se’ & Cd’ or Se’ & Cd’ & GSH. Se’ stands for Na_2_SeO_3_, Cd’ stands for CdCl_2_.

**
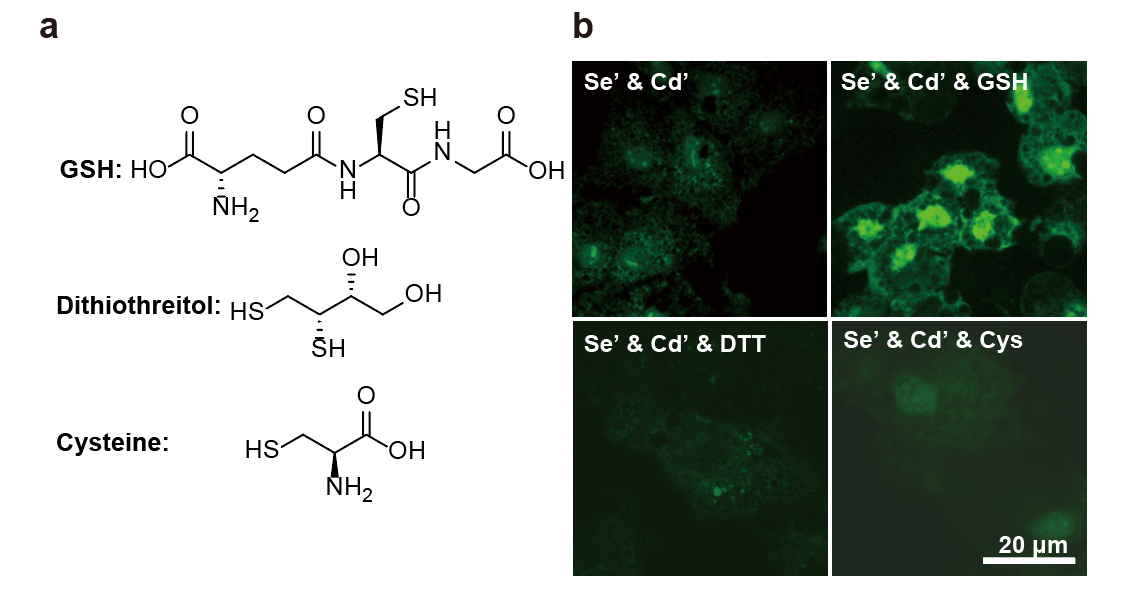
**

**Figure S5:** The effect of other reducing agents on QDs biosynthesis. **a)**The structures of GSH, and two common reducing agents dithiothreitol (DTT) and cysteine (Cys). **b)** Fluorescence images of Se’ & Cd’, Se’ & Cd’ & GSH-treated, DTT-treated, and Cys-treated cells. Se’ stands for Na_2_SeO_3_, Cd’ stands for CdCl_2_.


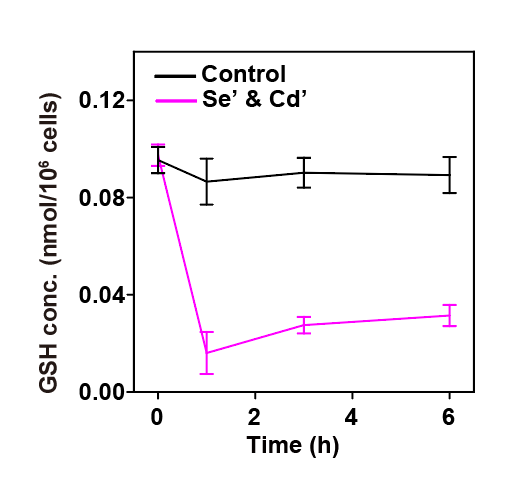


**Figure S6**: The concentration of GSH during cell selenization. Data represent the mean ± SD of three independent experiments. Se’ stands for Na_2_SeO_3_, Cd’ stands for CdCl_2_.


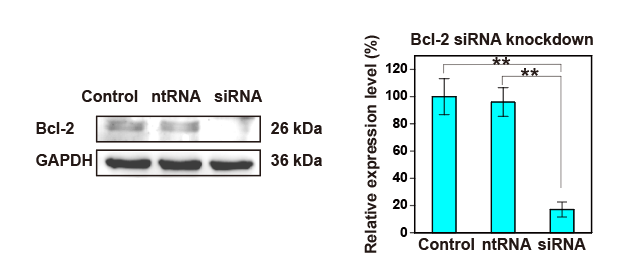


**Figure S7**: Translational inhibition of the Bcl-2 gene by siRNA knockdown as assessed by Western blotting. GAPDH was used for loading controls. Statistical significance was calculated with Student’s t test. **P* < 0.05, ***P* < 0.01, ****P* < 0.001.


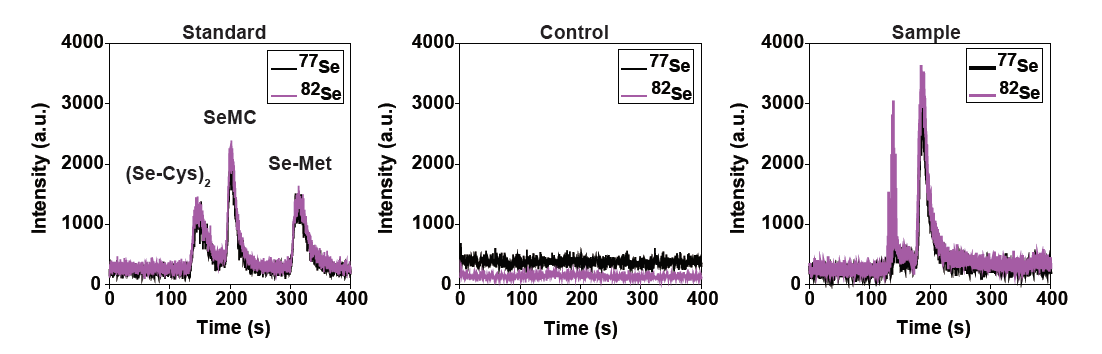


**Figure S8**: Characterization of intracellular reduced Se species in seleniumized MDCK cells. HPLC-ICP-MS chromatograms of **a**) Se species standards, **b**) control cells without Na_2_SeO_3_ treatment, and **c**) selenoamino acids isolated from seleniumized MDCK cells. The Se species standards were as follows: L-selenocysteine (Se-Cys)_2_, Se-methylseleno-L-cysteine (SeMC), and D, L-selenomethionine (Se-Met).


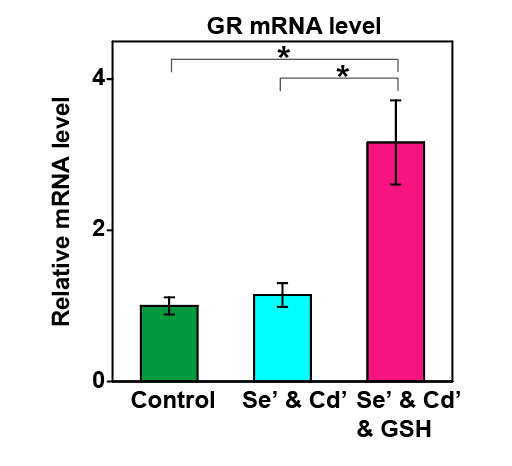


**Figure S9**: GR expression of control cells as well as cells treated with Se’ & Cd’ and Se’ & Cd’ & GSH. Se’ stands for Na_2_SeO_3_, Cd’ stands for CdCl_2_.


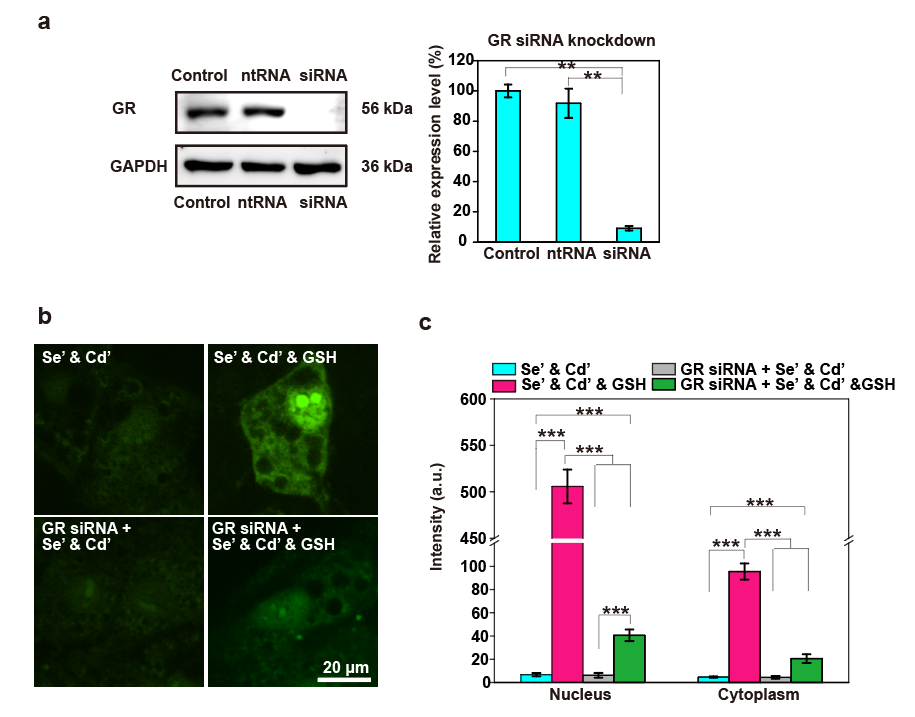


**Figure S10:** The GR gene knockdown data. **a**) Translational inhibition of the GR gene by siRNA knockdown as assessed by Western blotting. **b**) Confocal microscopy images of WT MDCK cells as well as GR knockdown cells synthesizing QDs with the treatment of Se’ & Cd’ and Se’ &Cd’ & GSH. **c**) Quantification of fluorescence intensity in the cell nucleus and cytoplasm. Imaging data represent means ± SD of triplicate measurements in multiple cells (*n* = 20 for **c**). Relative activity was calculated by normalizing density values against the maximal density for nucleus GR and whole-cell GR, respectively, after GAPDH correction. Statistical significance was calculated with Student’s t test. **P* < 0.05, ***P* < 0.01, ****P* < 0.001. Se’ stands for Na_2_SeO_3_, Cd’ stands for CdCl_2_.


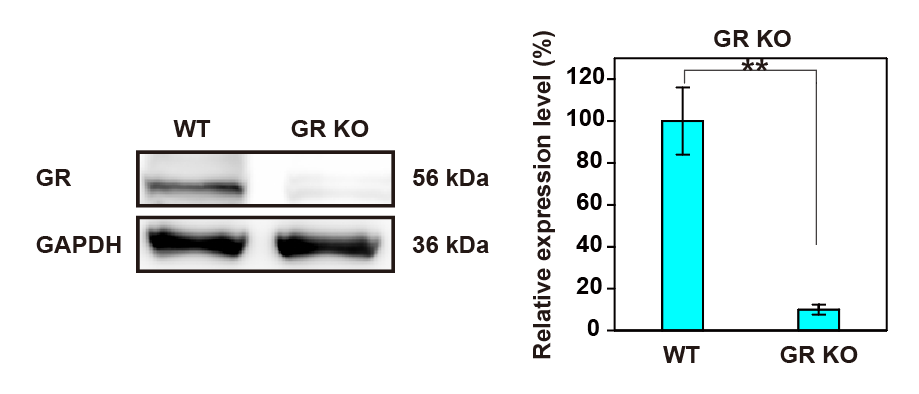


**Figure S11:** GR gene deletion in MDCK cells by CRISPR-Cas9 as assessed by Western blotting. GAPDH was used for loading controls. Statistical significance was calculated with Student’s t test. **P* < 0.05, ***P* < 0.01, ****P* < 0.001.


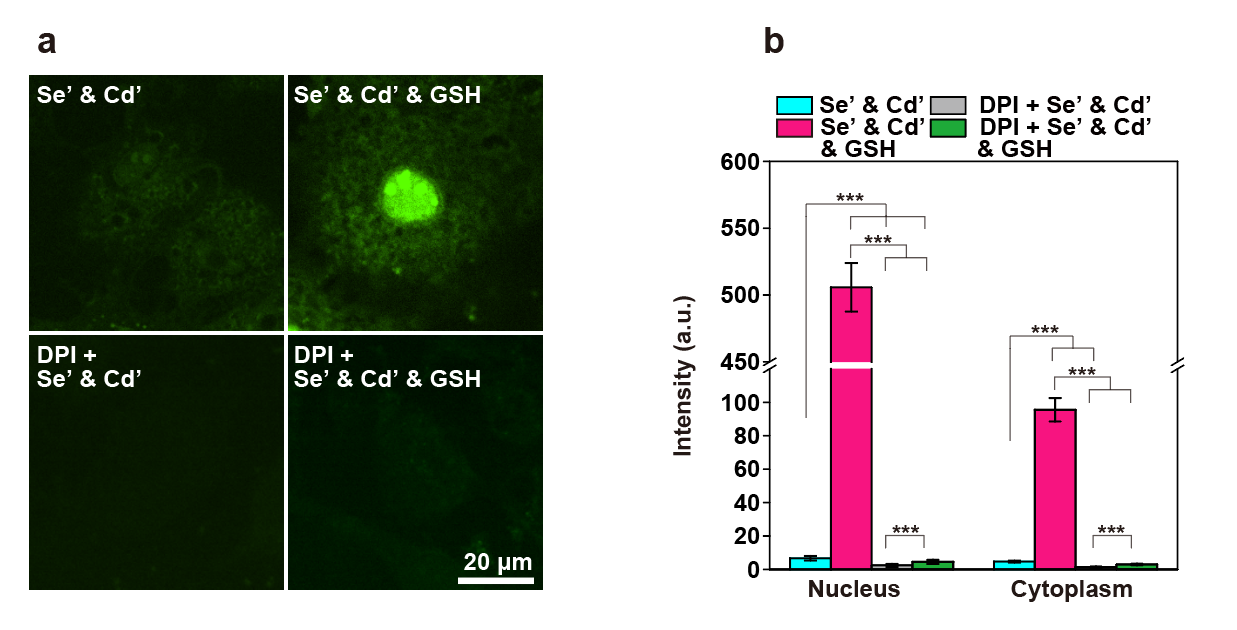


**Figure S12:** The effect of of DPI treatment on QDs biosynthesis**. a)** Control and DPI-treated cells biosynthesizing QDs with the treatment of Se & Cd and Se & Cd & GSH. **b**) Quantification of fluorescence intensity in **a**. Imaging data represent means ± SD of triplicate measurements in multiple cells (*n* = 20 for **a**). Statistical significance was calculated with Student’s t test. **P* < 0.05, ***P* < 0.01, ****P* < 0.001. Se’ stands for Na_2_SeO_3_, Cd’ stands for CdCl_2_.


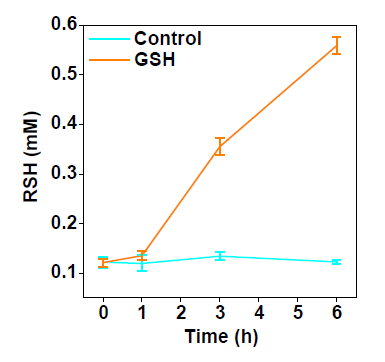


**Figure S13**: Time-dependent changes of RSH concentration after GSH addition.


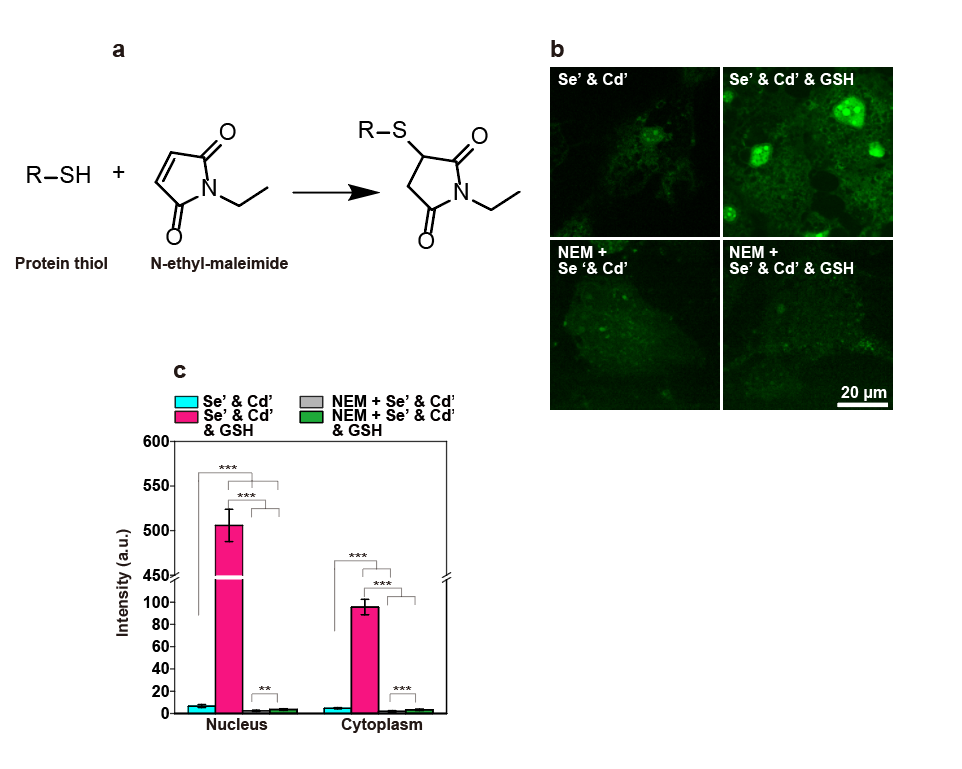


**Figure S14:** The effect of NEM treatment on QDs biosynthesis. **a**) NEM blocking of thiol groups inside the cell. **b**) Control and NEM-treated cells biosynthesizing QDs with the treatment of Se & Cd and Se &Cd & GSH. **c**) Quantification of fluorescence intensity in **b**. Imaging data represent means ± SD of triplicate measurements in multiple cells (*n* = 20 for **c**). Statistical significance was calculated with Student’s t test. **P* < 0.05, ***P* < 0.01, ****P* < 0.001. Se’ stands for Na_2_SeO_3_, Cd’ stands for CdCl_2_.


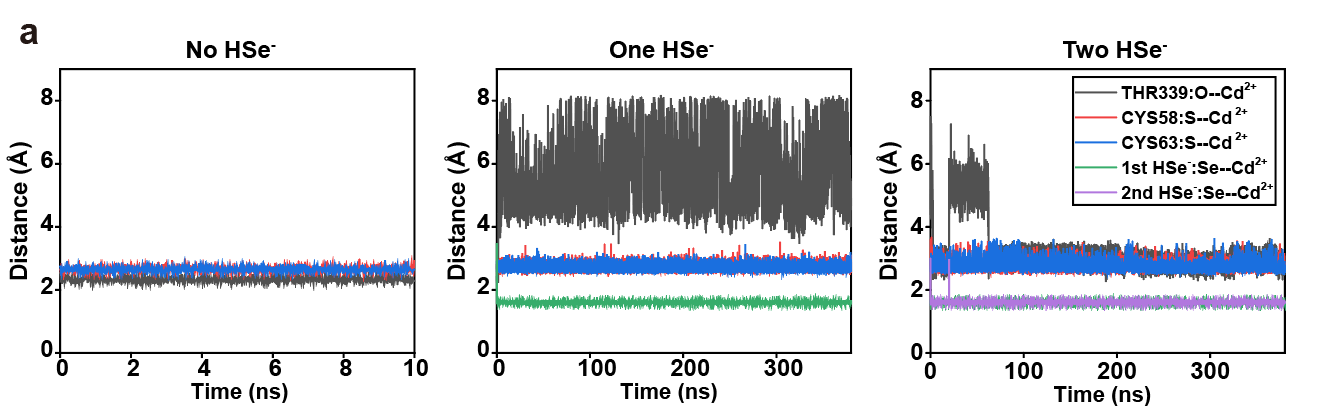


**Figure S15**: The distance between the ligands and the Cd^2+^ over time in 3 different models. **a)** Cys-Cd-Cys-Thr model. **b)** Cys-Cd-Cys-Thr model with one HSe^-^. **c)** Cys-Cd-Cys-Thr model with two HSe^-^.


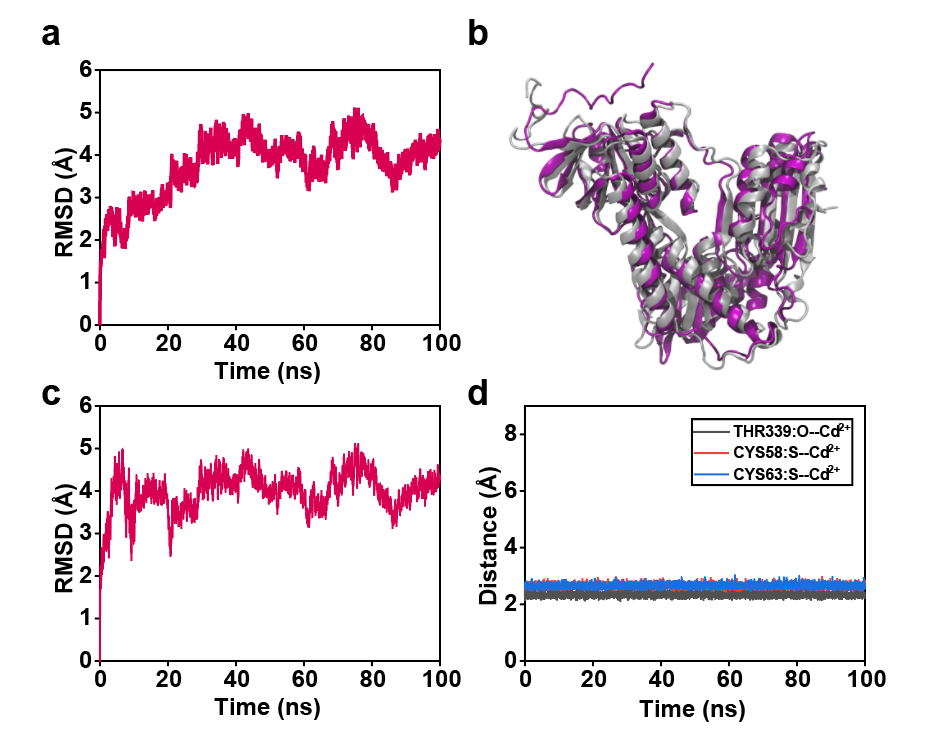


**Figure S16: a**) Cα root-mean-square deviation (RMSD) of the protein during 100-ns equilibration starting from the crystal structure, demonstrating that the system stabilizes within the 100-ns **b**) Comparison between the final structure of the simulation (purple) and the crystal structure (silver), indicating that there is no change in secondary structure. The fluctuations in the Cα RMSD are attributable to fluctuations of loops. **c**) Cα RMSD of the protein during 100-ns equilibration starting from the crystal structure with the addition of Cd^2+^. **d**) Distances between ligands and Cd^2+^ during the simulation. The simulation starting from the crystal structure with a manually added Cd^2+^ mirrors the initial simulation without Cd^2+^. The Cα RMSD in both cases fluctuates around 4Å. This suggests that the equilibration for the crystal structure with added Cd^2+^ does not introduce artificial effect.

**Table S1**: The binding energies of Cd^2+^ in different systems. * indicates that Cys-Cd-Cys-Thr has the strongest binding energy among the 6 systems.

| System | E_complex_(×10^5^ kcal/mol) | E_residue_ (×10^5^ kcal/mol) | E_Cd_(×10^5^ kcal/mol) | E_BSSE_(kcal/mol) | ΔE_bind_ (kcal/mol) |
| --- | --- | --- | --- | --- | --- |
| Cys-Cd | -6.3118 | -5.2607 | -1.0471 | 1.8778 | -394.2831 |
| Cys-Cd-Cys | -11.5744 | -10.521 | -1.0471 | 2.1183 | -625.9292 |
| Cys-Cd-His | -10.4905 | -9.4386 | -1.0471 | 1.5583 | -472.2033 |
| Cys-Cd-Cys-Thr | -15.0558 | -14.0008 | -1.0471 | 2.2639 | -786.5236* |
| GSH-Cd | -9.8684 | -8.8172 | -1.0471 | 2.0705 | -406.9165 |
| SeC-Cd | -18.8832 | -17.8322 | -1.0471 | 1.2598 | -389.615 |
